# Communicative And Affective Components in Processing Auditory Vitality Forms: An fMRI Study

**DOI:** 10.1093/cercor/bhab255

**Published:** 2021-08-25

**Authors:** G Di Cesare, V Cuccio, M Marchi, A Sciutti, G Rizzolatti

**Affiliations:** Italian Institute of Technology, Cognitive Architecture for Collaborative Technologies Unit, Genova, Italy; Department of Cognitive Science, Psychology, Education and Cultural Studies, University of Messina, Messina, Italy; Department of Computer Science, University of Milan, Milan, Italy; Italian Institute of Technology, Cognitive Architecture for Collaborative Technologies Unit, Genova, Italy; Istituto di Neuroscienze, Consiglio Nazionale delle Ricerche, Parma, Italy

**Keywords:** affective states, insula, interjections, language vitality forms, social communication

## Abstract

In previous studies on auditory vitality forms, we found that listening to action verbs pronounced gently or rudely, produced, relative to a neutral robotic voice, activation of the dorso-central insula. One might wonder whether this insular activation depends on the conjunction of action verbs and auditory vitality forms, or whether auditory vitality forms are sufficient per se to activate the insula. To solve this issue, we presented words not related to actions such as concrete nouns (e.g.,“ball”), pronounced gently or rudely. No activation of the dorso-central insula was found. As a further step, we examined whether interjections, i.e., speech stimuli conveying communicative intention (e.g., “hello”), pronounced with different vitality forms, would be able to activate, relative to control, the insula. The results showed that stimuli conveying a communicative intention, pronounced with different auditory vitality forms activate the dorsal-central insula. These data deepen our understanding of the vitality forms processing, showing that insular activation is not specific to action verbs, but can be also activated by speech acts conveying communicative intention such as interjections. These findings also show the intrinsic social nature of vitality forms because activation of the insula was not observed in the absence of a communicative intention.

## Introduction

Actions can be performed in different ways, known as vitality forms (Stern 2010). Actions, such as closing a door or shaking hands, can be carried out gently, neutrally or rudely. Emotions, such as joy or anger, can also be exhibited vehemently or mildly. Vitality forms reflect the agent’s affective state and play a crucial role in social interactions, both from the agent’s point of view and from the observer’s point of view. These forms of communication provide the observer with essential clues to navigate the social world and better understand others. In Stern’s (1985, 2010) view, actions, speech and, more generally, human behavior, are always characterized by vitality forms. The ability to express and understand vitality forms represents a fundamental component of human social interactions. In the absence of vitality forms, all actions would be similar and devoid of any affective color.

Research on infants has largely shown that, from the very beginning, vitality forms are important in pre-linguistic and linguistic interactions between children and their caregivers. Children tend to automatically attune to the attitudes of their caregivers expressed through their facial expressions (Meltzoff and Moore 1977; see also Schore 2021) as well as through the rhythm and tone of their voice (Marwick and Murray 2009).

In recent years, research has been conducted to identify the neural mechanisms underlying the ability to process vitality forms. In a series of fMRI studies, Di Cesare et al. (2013, 2015, 2016a, 2016b, 2019, 2020) found that the perception and the expression of actions performed with different vitality forms (such as rude or gentle) activate the dorso-central sector of the insula, more specifically the middle and posterior insula short gyri. Studies have shown that this region processes vitality forms and is endowed with the mirror mechanism (Rizzolatti and Craighero 2004; Iacoboni and Dapretto 2006; Fabbri-Destro and Rizzolatti 2008; Keysers and Fadiga 2008) that makes it possible for the observer to decode the vitality form of other people’s actions.

In addition to actions and gestures, human social interactions significantly rely on linguistic exchanges. In a previous study, Di Cesare et al. (2016b) demonstrated that listening to action verbs pronounced with gentle and rude vitality forms activated the parieto-fontal circuit and the dorso-central sector of the insula. The presentation of action verbs expressed by a robotic voice, and, therefore devoid of any vitality forms, activated the parieto-frontal circuit but not the insula, showing the specific role of the insula in the processing of vitality forms.

In light of these findings, it might be doubtful whether both vocal intonation (rude and gentle) and action-related meaning are necessary to activate the dorso-central insula.

Specifically, we wondered whether the activation of the insular cortex was due to the combination of vocal intonations (rude or gentle) and the action-related meaning of the verbs. Our previous studies showed that the action-related meaning per se was not sufficient to activate the insular cortex (as the insula was not activated by a robotic voice). However, these studies did not allow us to determine whether the action-related meaning of the action verbs was a necessary (although insufficient) condition for the activation of the insula.

To disentangle this issue, in the current study, we investigated whether listening to non-action-related single words, pronounced by a human voice with different vocal intonations (rude and gentle), is sufficient enough to activate the dorso-central insula. Furthermore, the current experimental design allowed us to assess whether, in addition to a vocal intonation (rude or gentle), the listened to words must have an identifiable communicative intention in order to activate the insular cortex. Consequently, we selected two types of stimuli: concrete nouns which, without a context, do not convey any social communicative intention [“palla” (ball); “chiave” (key); “birra” (beer); “gomma” (eraser); “tazza” (cup)], and interjections, which are uninflected words expressing a social communicative intention [“ciao” (hello); “grazie” (thanks); “prego” (you are welcome); “basta” (stop it); “scusa” (excuse me)]. Interjections are specific social acts (Searle 1969; see also Cuccio and Carapezza 2013) carried out with a single word that can be easily identified by the speaker even in the absence of a supporting context (for example, the act of greeting can be expressed by interjections such as “hello” or “bye”). As a control, we also presented a robotic voice pronouncing the same interjections and concrete nouns as the human actors but not conveying any vitality form.

If the dorso-central insula is activated by both concrete nouns and interjections, this would show that this insular sector is involved in the perception of vocal intonation per se, regardless of any action-related meaning. Another possibility is that, in addition to vocal intonation, a communicative intention should be present in the pronounced words. In the latter case, we expected that the dorso-central insula would be selectively activated by the interjections.

The results showed that the action-related meaning of the action verbs is not a necessary condition for the activation of the insula since the insular cortex was activated by interjections. Furthermore, our results also showed that vocal intonation (gentle and rude) is necessary but insufficient to activate the sector of the insula encoding vitality forms. Moreover, communicative intention is a necessary condition for activation of the dorso-central insula.

## Materials and Methods

### Participants

Sixteen participants took part in the experiment (nine females and seven males, mean age = 25.4, SD = 2). The choice to include 16 participants in the current study is based on results provided by a power analysis carried out on previous fMRI data concerning auditory vitality forms (Di Cesare et al. 2017). The power analysis results indicated that, in order to obtain a large effect in the left dorso-central insula due to auditory vitality forms, it is essential to collect a sample consisting of at least 12 participants [effect size dz = 1.18, α = 0.05, β = 0.95]. All participants gave their written informed consent to participate in the experiment. The experiment was approved by the ethics committee of the University of Parma (552/2020/SPER/UNIPR) in accordance with the Declaration of Helsinki. All participants had normal or corrected-to-normal vision.

### Stimuli

**Figure 1:**
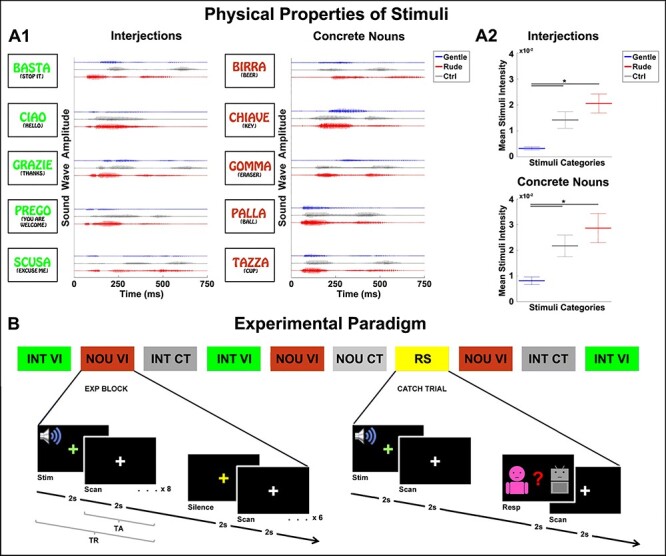
Physical characteristics of the audio stimuli and the experimental paradigm. Graph A1 shows the sound wave amplitude relative to the interjections and concrete nouns pronounced both by a male actor with different vocal intonations (gentle, blue color; rude, red color) or by a robotic voice (control, gray color). Graph A2 shows the mean intensity of audio stimuli pronounced by the male actor and the robotic voice. Asterisk (^*^) indicates a significant difference among the conditions (gentle, rude, control) revealed by the post-hoc analysis (*P* < 0.05 Newman Keuls correction). Image shows the experimental paradigm adopted in the experiment (B). Audio stimuli were presented in blocks of eight consecutive stimuli (duration 32 s; 8 TR) of the same condition (*interjections vocal intonation:* INT VI; *concrete nouns vocal intonation (VI)*: NOU VI; *interjections control*: INT CT; *concrete nouns control:* NOU CT) followed by a silent period lasting 24 s (6 TR). In 30% of cases, the catch trial blocks (response, RS) were randomly presented in which the participants had to indicate the agent’s voice of the last presented stimulus by pressing a button on a response box placed inside the scanner (male voice, female voice, robotic voice).

Native Italian participants were presented with audio stimuli consisting of two distinct categories of Italian words: interjections and concrete nouns. In particular, two actors (a male and a female) pronounced 5 different interjections [“ciao” (hello); “grazie” (thanks); “prego” (you are welcome); “basta” (stop it); “scusa” (excuse me)] and 5 different concrete nouns [“palla” (ball); “chiave” (key); “birra” (beer); “gomma” (eraser); “tazza” (cup)]. All the words were pronounced by the actors using two different vocal intonations: rude and gentle (vitality forms condition). There words were pronounced by the female actress in 50% of the cases and by the male actor in the other 50% of the cases. Additionally, as the control, we also presented a robotic voice pronouncing the same interjections and concrete nouns as the actors. This robotic voice pronounced the same words maintaining the meaning but not conveying a vocal intonation, thus allowing us to control the effect of vocal intonation for both interjections and concrete nouns pronounced by the human voice. This control voice was obtained by a vocal synthesizer (TextAloud software) and then processed with FL Studio 11 software (see Fig. 1). Subsequently, each robotic word (interjections and concrete nouns) was equated for loudness in order to match the mean value loudness of the corresponding words pronounced by the two actors with different vocal intonations (rude and gentle) (see Fig. 1*A*2).

All the words pronounced by the actors were recorded using a cardioid condenser microphone (RODE NT1) placed 20 cm from the speaker and digitized with an A/D converter module with a phantom power supply (M-AUDIO M-TRACK). The audio stimuli were then processed with FL Studio 11 software. Most importantly, the audio stimuli recorded with vocal intonations (rude and gentle) maintained their ecological loudness. A total of 40 experimental audio stimuli (5 interjections + 5 concrete nouns × 2 vitality forms × 2 actors) and 10 control stimuli (5 robotic interjections + 5 robotic concrete nouns) were presented. Each audio stimulus was presented in a time window of 2 s.

The physical characteristics of all the presented audio stimuli were assessed using MATLAB (The MathWorks, Natick, MA, USA). For each audio stimulus, we estimated the sound wave amplitude and the pitch for all four conditions (interjections vocal intonation [INT VI], concrete nouns vocal intonation [NOU VI], interjections control [INT CT], concrete nouns control [NOU CT]). Figure 1*A*1 shows the physical characteristics related to both the interjections and the concrete nouns pronounced by the male actor (see Supplementary Figure S1 for the words pronounced by the female actor).

Finally, to obtain the frequency of each word, a lexical database for Italian words was used (www.ge.ilc.cnr.it/lessico.php). The parameter of lemma frequency was obtained for both word categories (interjections: mean frequency = 0.47, SD = 0.26; concrete nouns: mean frequency = 0.74, SD = 0.12). Moreover, the words were also matched across conditions for the length of the letters (interjections: mean length = 5 letters, SD = 0.7; concrete nouns: mean length = 5.2 letters, SD = 0.44) (for details see Table 1).

**Table 1:** Lemma frequency (Bambini and Trevisan 2012) and number of characters for each verb

Stimuli	Frequency	Length
**Interjections**		
Basta (Stop it)	0.75	5
Ciao (Hello)	0.47	4
Grazie (Thanks)	0.72	6
Prego (You are welcome)	0.24	5
Scusa (Excuse me)	0.17	5
Mean (SD)	0.47 (0.26)	5 (0.7)
**Concrete nouns**		
Birra (Beer)	0.66	5
Chiave (Key)	0.91	6
Gomma (Eraser)	0.83	5
Palla (Ball)	0.72	5
Tazza (Cup)	0.6	5
Mean (SD)	0.74 (0.12)	5.2 (0.44)

### Experimental Design

A sparse block design (van Atteveldt et al. 2004; Gazzola et al. 2006) was used in the experiment. The scan cycle (TR) was composed of 37 sequential slices (slice thickness = 3 plus inter-slice gap = 0.5 mm) covering the entire brain collected in 2 s (acquisition time) followed by a period of silence lasting 2 s (TR = 4 s). In the experiment, four conditions were presented: INT VI, NOU VI, INT CT and NOU CT. The experimental stimuli were presented in blocks of eight consecutive stimuli of the same condition (duration 32 s; 8 TR; see Fig. 1*B*) followed by a period of silence lasting 24 s (6 TR) (Fig. 1*B*). Each audio stimulus was presented during the period of silence. The experiment was composed of two functional runs with a total of four blocks (32 single trials) for each condition, presented randomly. Each functional run lasted about 10 min.

### Paradigm and Task

The participants laid in the scanner in a dimly lit environment. The stimuli were presented via a digital audio system with 30 dB noise-attenuating headset with a 40–40 kHz frequency response (VisuaSTIM). E-Prime 2 Professional software was used to present the stimuli presentation and record the participants’ answers. Before the experiment, the participants, already lying in the scanner, performed a training session consisting of a random presentation of all the audio stimuli to ascertain their ability to recognize both the interjections and concrete nouns stimuli (94% on average). During the stimuli presentation, the participants were requested to fixate on a green cross on a black screen and listen to the audio stimuli paying attention to the agent’s voice. The experimental stimuli were presented in blocks (eight consecutive stimuli of the same condition), and the catch trial blocks were intermixed with the experimental blocks. During the random presentation of the catch trial blocks, the participants were required to indicate the agent’s voice from the previous presented stimulus by pressing a button (male voice, female voice, robotic voice) (catch trial, Fig. 1*B*) (Have you listened a female voice or a robotic voice?). The catch trials lasted 2 s. The analysis of the catch trials showed that, during the experiment, the participants’ mean response accuracy was 96%.

### fMRI Data Acquisition

Anatomical T1-weighted and functional T2^*^-weighted MR images were acquired with a 3 Tesla General Electric scanner equipped with an 8-channel receiver head-coil. Functional images were acquired using a T2^*^-weighted gradient-echo, echo-planar (EPI) pulse sequence acceleration factor asset 2, 37 sequential transverse slices (slice thickness = 3 plus inter-slice gap = 0.5 mm) covering the entire brain, with a TR time of 4000 ms (TE = 30 ms, flip-angle = 90 degrees, FOV = 205 × 205 mm^2^, in-plane resolution 2.5 × 2.5 mm^2^). The scanning sequence consisted of 140 ascending sequential volumes. Additionally, a T1-weighted structural image was acquired for each participant (acceleration factor arc 2, 156 sagittal slices, matrix 256 × 256, isotropic resolution 1 × 1 × 1 mm^3^, TI = 450 ms, TR = 8100 ms, TE = 3.2 ms, flip angle 12°).

### Statistical Analysis

Data analysis was performed with SPM12 (Wellcome Trust Centre for Neuroimaging, London, UK). The first three volumes of each run were discarded to allow for the T1 equilibration effects. For each participant, the functional volumes were first slice timing corrected according to the sparse imaging acquisition (TA: acquisition time = 2000 ms), realigned to the mean volume and unwarped for between-scan motion correction. Subsequently, the T1-weighted image was resampled into the functional image space before segmentation into gray, white and cerebrospinal fluid, and normalization to the Montreal Neurological Institute (MNI) space, according to SPM12 pre-processing pipeline. Finally, the spatial transformations derived from the segmentation step were then applied to the realigned EPIs for normalization to the MNI space with a voxel size of 2 × 2 × 2 mm. At the end of pre-processing, all the functional normalized volumes were spatially smoothed with a 6-mm full-width half-maximum isotropic Gaussian kernel. For all participants, head motion was carefully checked along the x (pitch movement), y (yaw movement) and z (roll movement) directions; none of the participants met the exclusion criteria of 3 mm mean displacement. Data were analyzed using a random-effects model (Friston et al. 1999), implemented in a two-level procedure. In the first level, single-subject fMRI BOLD signal was modeled in two General Linear Models (GLMs) by a design-matrix comprising the onsets, the durations of each event according to the experimental task for each functional run. The first GLM model was composed of five regressors: interjections vocal intonation, concrete nouns vocal intonation, interjections control, concrete nouns control and response. The second GLM model was composed of the same five regressors plus two contrasts: interjections vocal intonation versus interjections control and concrete nouns vocal intonation versus concrete nouns control. Audio stimuli were presented in blocks of eight consecutive stimuli of the same condition (interjections vocal intonation: rude and gentle; concrete nouns vocal intonation: rude and gentle; interjections control, concrete nouns control). Within each block, the audio stimuli were modeled as a single event lasting 2 s. The response was also modeled as a single event lasting 2 s.

In the second-level analysis (group analysis), for each participant, the corresponding contrast images of the first level were entered into two flexible ANOVA with sphericity-correction for repeated measures (Friston et al., 2002). The first model (first group analysis) was composed of four regressors; it considered both the activation patterns obtained for different conditions (interjections vocal intonation, concrete nouns vocal intonation, interjections control, concrete nouns control) and the activations resulting from the direct contrast between the conditions (interjections vocal intonation vs interjections control, concrete nouns vocal intonation vs concrete nouns control, interjections vocal intonation vs concrete nouns vocal intonation, interjections control vs concrete nouns control). The second model (second group analysis) was composed of two regressors (interjections vocal intonation vs interjections control, concrete nouns vocal intonation vs concrete nouns control); it considered the activations between each condition versus the control. The location of the activation foci was determined in the stereotaxic space of the MNI coordinates system.

### Testing for the Interjections Effect: Region-of-Interest Analysis

To assess the specificity of the brain areas highlighted from the contrast interjections vocal intonation versus concrete nouns vocal intonation, we also extracted the BOLD signal change for each participant relative to statistical maps obtained in the first group analysis relative to all the regressors (interjections vocal intonation, concrete nouns vocal intonation, interjections control, concrete nouns control). In this region-of-interest (ROI) analysis, to avoid the problem of circularity, statistical tests were not applied (Kriegeskorte et al. 2009). Moreover, to examine whether the effect highlighted in the dorso-central insula from the contrast interjections vocal intonation versus concrete nouns vocal intonation was specific for the word type, we conducted a ROI analysis. Specifically, using the SPM Rex Toolbox (http://web.mit.edu/swg/rex), for each participant, we extracted the BOLD signal change relative to statistical maps obtained in the second group analysis from the following contrasts: interjections vocal intonation versus interjections control and concrete nouns vocal intonation versus concrete nouns control.

## Results

### Overall Effect of the Interjections, Concrete Nouns and Controls

**Figure 2:**
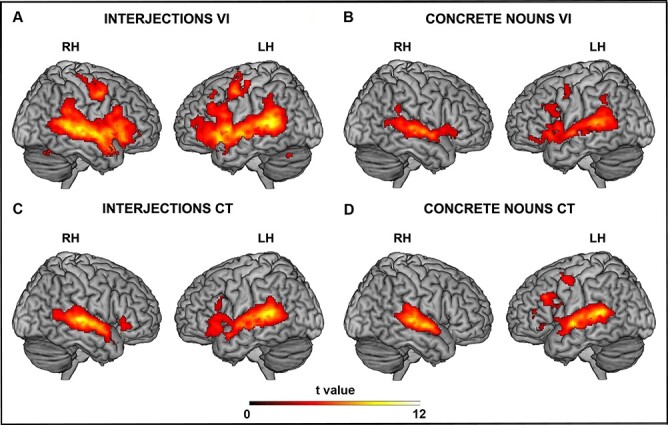
Brain activations resulting from listening to interjections vocal intonation (*A*), concrete nouns vocal intonation (*B*), interjections control (*C*), concrete nouns control (*D*), versus baseline. These activations are rendered using a standard Montreal Neurological Institute brain template (P_FWE_ < 0.05 cluster level). LH: left hemisphere; RH: right hemisphere.

Hearing interjections vocal intonation expressed by actors’ voices revealed a BOLD signal increase in the participants’ auditory areas of the superior temporal gyrus, left ventral premotor cortex, left prefrontal cortex and the inferior frontal gyrus in both hemispheres (Fig. 2*A*). Furthermore, there was bilateral activation of the insula. Listening to the interjections control activated the auditory temporal areas bilaterally and resulted in a rather weak activation of the left inferior frontal gyrus (Fig. 2*C*). Moreover, listening to concrete nouns vocal intonation produced a weak signal increase in the auditory temporal areas bilaterally, the left premotor cortex and the left inferior frontal gyrus (Fig. 2*B*; for statistical values and coordinates see Table 2A–C). Finally, listening to concrete nouns control activated a pattern that was very similar to the one obtained for the concrete nouns vocal intonation condition (see Fig. 2*D*).

**Table 2:** Brain activations obtained during the listening of interjections VI (A); concrete nouns VI (B); interjections CT (C); concrete nouns CT (D); brain activation resulting from the contrasts interjections VI versus interjections CT (E); and interjections VI versus concrete nouns VI (F)

Contrast of interest	Left hemisphere					Right hemisphere			
	*x*	*y*	*z*	*Z*-score		*x*	*y*	*z*	*Z*-score
**(A) Interjections VI** versus **baseline**									
Middle temporal gyrus	−62	−36	8	7.74	Superior temporal gyrus	50	−22	−6	Inf
IFG	−50	18	−10	6.92	Posterior-medial frontal cortex	4	8	66	6.75
Insula	−34	6	2	5.13	PMC	54	0	44	6.06
Posterior-medial frontal cortex	−4	4	62	5.95	Cerebellum	32	−58	−28	5.26
Putamen	−24	−2	10	4.80	Insula	38	10	0	4.74
Thalamus	−12	−8	12	4.43					
Calcarine gyrus	−4	−84	−2	3.71					
**(B) Concrete nouns VI** versus **baseline**									
Middle temporal gyrus	−62	−36	8	6.63	Superior temporal gyrus	50	−22	−6	6.73
Superior temporal gyrus	−58	−10	4	5.84					
Posterior-medial frontal cortex	−6	8	54	5.46					
IFG	−46	28	−6	4.79					
PMC	−44	2	44	4.70					
**(C) Interjections CT** versus **baseline**									
Middle temporal gyrus	−62	−36	8	7.66	Superior temporal gyrus	58	−24	2	Inf
**(D) Concrete nouns CT** versus **baseline**									
Superior temporal gyrus	−62	−38	10	7.74	Superior temporal gyrus	60	−24	2	7.09
IFG	−50	28	30	5.10					
**(E) Interjections VI** versus **interjections CT**									
IFG	−46	−10	2	4.79	IFG	52	12	0	5.29
Insula	−44	10	0	4.49	Calcarine gyrus	4	−82	4	5.12
					Superior temporal gyrus	50	−22	−6	5.04
					pSTS	56	−40	6	4.67
					Posterior-medial frontal cortex	8	12	54	4.84
**(F) Interjections VI** versus **concrete nouns VI**									
Insula	−42	8	0	3.98	PMC	56	0	44	4.50
					pSTS	56	−38	4	4.36
					aSTS	54	4	−20	4.19
					Lyngual gyrus	2	−82	4	3.95
					Frontal operculum/IFG	52	4	6	3.79

**Figure 3:**
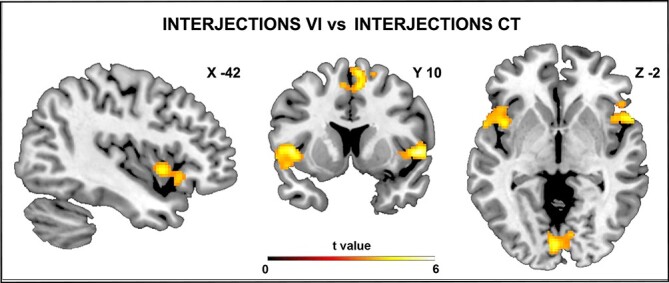
Brain activations (*A*) obtained from the contrast *interjections vocal intonation* versus *interjections control.* These brain activations are rendered using a standard Montreal Neurological Institute brain template (*P*_FWE_ < 0.05 at cluster level). LH: left hemisphere; RH: right hemisphere.

**Figure 4:**
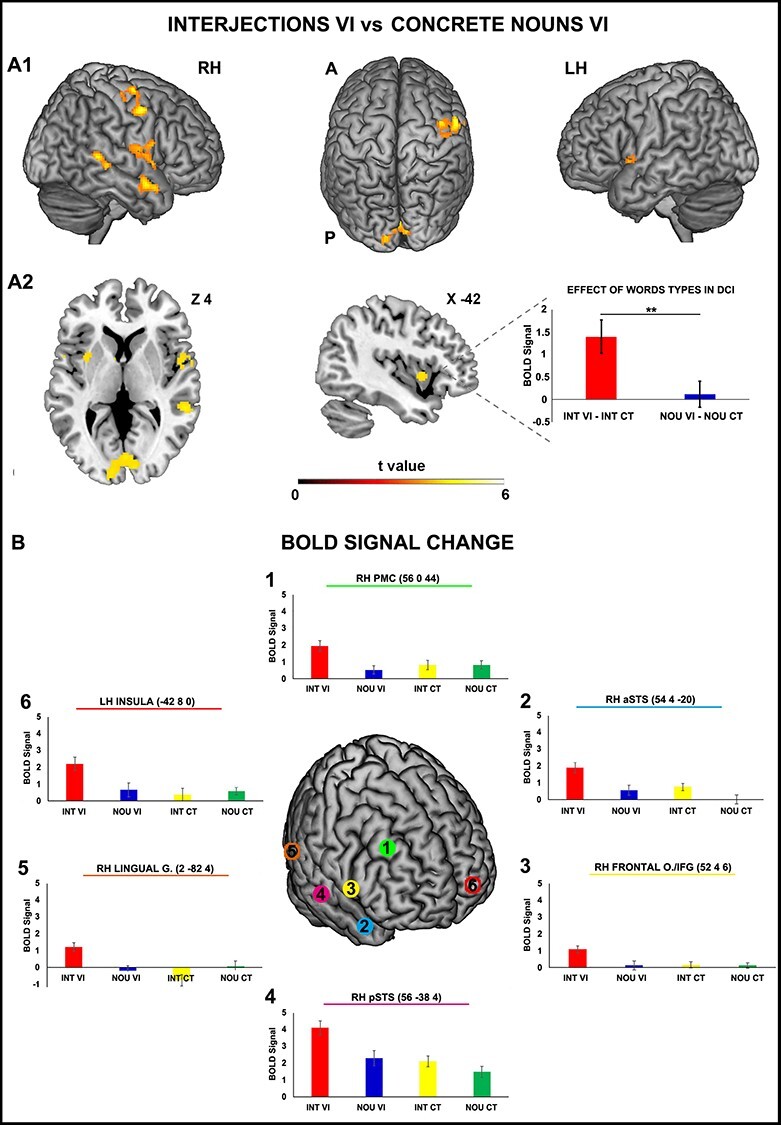
Brain activations resulting from the contrast *interjections vocal intonation* versus *concrete nouns vocal intonation* (*A*1). The parasagittal sections show the activation of the right premotor cortex, the right frontal operculum, the right anterior and posterior part of the superior temporal sulcus, the right lingual gyrus and the left dorso-central insula (*A*2). These brain activations are rendered using a standard Montreal Neurological Institute brain template (P_FWE_ < 0.05 at the cluster level). The bar graphs indicate the BOLD signal change extracted from the dorso-central insula during the contrasts *interjections vocal intonation* versus *interjections control and concrete nouns vocal intonations* versus *concrete nouns control* (A2, right side panel). The BOLD signal change in brain areas resulting from the *contrast interjections vocal intonations* versus *concrete nouns vocal intonations* (*B*). The vertical lines indicate the standard error of the means. ^*^P < 0.05, ^*^^*^P < 0.01.

Local maxima, as shown in Figures 2–4, are given in MNI standard brain coordinates; significant threshold is set at *P*_FWE_ < 0.05 (cluster level)

### Contrasts between Vocal Intonations and Controls

The direct contrast vocal intonations versus controls (interjections vocal intonation and concrete nouns vocal intonation vs interjections control and concrete nouns control) enhanced the activation of the insula and the middle cingulate cortex bilaterally as well as the right middle temporal gyrus and the left cerebellum. Additionally, the direct contrast interjections vocal intonation versus interjections control revealed a significant activation pattern in the left insular cortex extending to the inferior frontal gyrus, in the left thalamus, in the right posterior-medial frontal cortex, the right inferior frontal gyrus, the posterior part of the right superior temporal sulcus and the right calcarine gyrus (Fig. 3). The direct contrast concrete nouns vocal intonation versus concrete nouns control did not reveal a significant activation pattern in either the left hemisphere or the right hemisphere. Finally, the direct contrast interjections vocal intonation versus concrete nouns vocal intonation produced activations in the left dorso-central insula, the right ventral precentral gyrus, the right frontal operculum extending to the inferior frontal gyrus, the right anterior and posterior parts of the superior temporal sulcus and the lingual gyrus, bilaterally (Fig. 4*A*1 and *A*2; for statistical and coordinates see Table 2D and E). No significant activations were observed with the opposite contrast (concrete nouns vocal intonation vs interjections vocal intonation).

The interaction between the stimulus type (interjections, concrete nouns) and the vocal intonations (human voice: gentle and rude; control: robotic voice) activates the lingual gyrus. Finally, the results of the ROI analysis conducted on the left dorso-central insula revealed a significant difference in the BOLD signal (paired sample *t*-test, *P* < 0.01) between the following contrasts: interjections vocal intonation versus interjections control and concrete nouns vocal intonation versus concrete nouns control. Specifically, this result indicates that listening to interjections pronounced with vocal intonations in comparison with the control produced a higher activation than listening to concrete nouns pronounced with vocal intonations in comparison to the control (see Fig. 4*A*2, right panel).

## Discussion

In previous fMRI experiments, we demonstrated that listening to action verbs pronounced by a human voice with gentle or rude vocal intonation activated the dorso-central insula encoding vitality forms in comparison to the neutral control (robotic voice) (Di Cesare et al. 2016b, 2018). Are both the vocal intonation and the action-related meaning of the verbs necessary to activate the dorso-central insula? Our previous data showed that the action-related meaning per se is insufficient to activate the insular cortex. The words pronounced by the robot, although understood by the participants, did not activate the insula.

Therefore, it appears that vocal intonation is a fundamental factor for insular activation. However, these previous results did not allow us to determine whether the action-related meaning of the verbs was also a necessary (although not sufficient) condition for activation of the insula.

In the present study, we attempted to answer this question by testing whether the insular activation could be generalized to listening to other types of words, such as interjections or concrete nouns. Specifically, we addressed this issue by presenting single concrete nouns (beer, ball, etc.) and interjections (thank you, hello, etc.) expressed by human actors either gently/rudely or by a robotic voice pronouncing the same words without vocal intonation (control). Our hypothesis was that regardless of whether vocal intonation was enough to activate the insula, listening to these words (concrete nouns and interjections) pronounced with gentle and rude vocal intonations should be sufficient to trigger it, as the action verbs did in our previous study.

In this experiment, we also addressed a related issue, which is whether, in addition to vocal intonation, the presence of a communicative intention is also necessary to activate the insula (Cuccio 2012; Cuccio et al. 2014; Carapezza and Cuccio 2018). If this was the case, only interjections (thank you, please etc.), which were pronounced in different ways (gently or rudely) and which simultaneously conveyed communicative intentions, should activate the insular cortex. Our data showed that listening to concrete nouns (beer, ball, etc.) pronounced gently and rudely, in comparison to the control stimuli, did not produce a significant activation of the insular cortex. These data clearly show that vocal intonation per se is insufficient to activate the dorso-central insula. In contrast, listening to interjections, pronounced rudely or gently, which conveyed a communicative intention, activated the dorso-central insula, as was the case for the action verbs used in our previous experiments (Di Cesare et al. 2015, 2018).

In summary, although both interjections and concrete nouns were characterized by an affective component (rude and gentle vocal intonation), there was a striking difference in brain activations between these word categories. This difference seems to rely on the fact that only the interjections clearly conveyed a social intention and were communicatively meaningful to the participants in our study. In this respect, the interjections were very similar to the action verbs pronounced in the imperative mood that we used in our previous studies, such as “take it,” “give me,” etc. Both those actions verbs and the interjections used in the current study expressed a communicative action that was easily identifiable by the participants.

Thus, our data clearly showed that the left dorso-central insula is not automatically activated by vocal intonations per se since this area was not recruited by the processing of concrete nouns (which refer to concrete entities that imply hand-object interaction) pronounced by a human voice with gentle and rude vocal intonations. Furthermore, this insular sector was never activated by the processing of both interjections and concrete nouns when these were pronounced by a robotic voice, excluding that the physical properties of the acoustic stimuli could explain the vitality form effect.

The results of the ROI analysis allowed us to better understand the role of the word categories (interjections vs concrete nouns) in the activation of the dorso-central insula. This analysis showed that interjections produced enhanced activation of this cortical area, suggesting that this brain sector is involved in decoding words conveying social communicative intentions, such as interjections.

Taken together, these findings demonstrate that insular activation is not specific to action verbs that have vocal intonation; it can be generalized to other types of words, such as interjections. The results also show that an identifiable communicative intention, in addition to vocal intonation, is necessary to activate the sector of the left insula encoding vitality forms since vocal intonation per se is not sufficient.

To better understand our results, it might be useful to consider them in the general framework of insular functions. We know that, based on different sensory inputs (homeostatic, visceral, nociceptive and somatosensory inputs) (see Craig 2002; Damasio and Carvalho 2013), the insula is considered to generate a representation of the internal state of the body in which somatic and visceral components are integrated and ultimately give rise to a “feeling of the body” (see Singer et al. 2004).

In a meta-analysis based on 1768 fMRI studies in humans, Kurth et al. (2010) reported an insular organization consisting of four distinct functional fields: the cognitive field, the sensorimotor field, the olfactory-gustatory field and the socio-emotional field. The sensorimotor field appears to closely correspond to the sector involved in the processing of vitality forms. Interestingly, previous results suggested that the processing of vitality forms is related to action perception/expression (Di Cesare et al. 2015, 2018). The current study’s findings extend this view to linguistic actions. The latter are words expressing a social action, such as apologizing or greeting someone (Searle 1969; Cuccio and Fontana 2017).

As determined in our study, listening to interjections relative to concrete nouns, activated the left dorso-central insula and the circuit in the right hemisphere comprising the posterior part of superior temporal sulcus (pSTS), the anterior part of the superior temporal sulcus (aSTS), the inferior frontal gyrus (IFG) and the premotor cortex (PMC). These data are fully in agreement with Sammler et al. (2015), who identified this same brain network during a prosody perception task. In their fMRI study, Sammler et al. (2015) asked participants to listen to linguistic stimuli (the words “pear” and “bear”) pronounced with different a rhythm and tone such that they conveyed two different communicative intentions (naming and asking). In that study, the participants were requested to determine whether the speaker was naming or asking for the object; in a control condition, the same words were pronounced with no prosody and the participants were asked to determine whether the speaker said “bear” or “pear.” Sammler et al. (2015) found that the right hemisphere network comprising pSTS, aSTS, IFG and PMC was only activated under the experimental condition.

In light of these results, one might wonder why, in the present study, we found the activation of this network during the processing of interjections pronounced with different vocal intonations, whereas the same network was not activated during the processing of concrete nouns. A review of neuroimaging studies on the neural bases of prosody (Paulmann 2016) suggested that the neural network underlying the processing of prosodic clues spans both hemispheres. Furthermore, and more importantly, previous studies have suggested that various factors impact the lateralization and recruitment of areas for the perception of prosody. Specifically, Paulmann (2016) highlighted that this may depend on many factors, such as task demands (high/low; Plante et al. 2002) and stimulus type (syllable/word/sentence; Gandour et al. 2004; Meyer et al. 2002).

Our study differed significantly from Sammler et al. (2015) with regards to both task demands and stimuli properties. As for the task, while Sammler et al. (2015) asked the participants to decide whether the stimuli they heard could be interpreted as naming or requesting, we did not ask to our participants to express any judgment. In our study, the participants were invited to listen to the stimuli while lying in the scanner. As for the prosodic clues, in Sammler et al. (2015), the stimuli reproduced the prosody associated with specific and very familiar communicative actions (naming and requesting). In contrast, the prosody in our stimuli did not reproduce the rhythm and tone associated with specific communicative actions. Thus, in Sammler et al.’s (2015) study, the activation of this right hemisphere network subserving the perception of prosody seems to be specifically linked to the understanding of the speaker’s intentions characterizing different communicative actions (see also Hellbernd and Sammler 2016, 2018).

As anticipated, prosody in our stimuli did not reproduce the rhythm and tone usually associated with familiar communicative actions. Furthermore, by using a more ecological task (participants were only instructed to listen to the stimuli), we did not force them to interpret those stimuli in terms of a communicative action. However, the combination of interjections with different vocal intonations ecologically evoked a communicative action. Only this combination (vocal intonations and communicative intentions), which in our stimuli was exclusively present in the interjections, determined the activation of the left dorso-central insula and the right hemisphere network. These areas were not activated by the processing of concrete nouns, although those words were pronounced with the same vocal intonations as the interjections. Thus, it is plausible to think that the difference between concrete nouns and interjections may be due to the fact that the concrete nouns were not communicatively meaningful for the participants as they did not convey a communicative intention.

In summary, the activation of the left dorso-central insula during the perception of auditory vitality forms is not related only to action-related verbs since the same area was found to be activated during the processing of interjections pronounced with different vocal intonations. The activation of this insular sector is not automatic; thus, it is not merely determined by the physical properties of the stimuli since the left insula was not activated by the concrete nouns pronounced with different vocal intonations (gentle and rude). In the present study, the activation of the left insula and the right hemisphere network during the processing of interjections is dependent on the combination of two components: a communicative intention and the vocal intonation. The former refers to the expression of a social action; the latter refers to the expression of an affective component displayed by the linguistic stimuli.

In conclusion, since insular activation was observed only during the encoding of interjections, which were perceived as a communicative act, our findings enlarge the understanding of the processing of vitality forms. Vitality forms are always related to the perception/expression of social actions, which can be carried out in the motor dimension or the linguistic dimension. The combination of a communicative intention, conveyed by interjections, and the affective component, conveyed by vocal intonations, contributed to the representation of vitality forms in the receiver.

## Funding

Giuseppe Di Cesare and Alessandra Sciutti are supported by a Starting Grant from the European Research Council (ERC) under the European Union’s Horizon 2020 research and innovation programme. G.A. No. 804388, wHiSPER. Giacomo Rizzolatti was supported by a grant Lombardia è Ricerca from Lombardia region.

## Notes

*Conflict of Interest:* None declared.

## Supplementary Material

Figure_S1_bhab255

FIGURE_S1_bhab255
