# Supplementary material for: Communicative And Affective Components in Processing Auditory Vitality Forms: An fMRI Study
Source: Cereb Cortex. 2021 Aug 25;32(5):909–18. doi: 10.1093/cercor/bhab255 (PMC8889944; doi:10.1093/cercor/bhab255)
Supplement: Figure_S1_bhab255 [file figure_s1_bhab255.docx]

Figure S1: Graph shows the wave sounds of vocal stimuli (A) and their mean intensity (B). The stimuli were pronounced by the female actress and the robotic voice.
