# Supplementary figures and images for: Communicative And Affective Components in Processing Auditory Vitality Forms: An fMRI Study
Source: Cereb Cortex. 2021 Aug 25;32(5):909–18. doi: 10.1093/cercor/bhab255 (PMC8889944; doi:10.1093/cercor/bhab255)

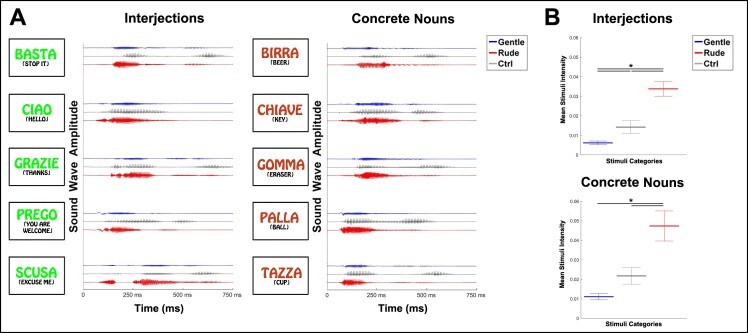

Supplement: FIGURE_S1_bhab255 [file figure_s1_bhab255.jpg]
